# Expanding the application of anti-CRISPR proteins in plants for tunable genome editing

**DOI:** 10.1093/plphys/kiad076

**Published:** 2023-02-09

**Authors:** Yang Liu, Guoliang Yuan, Brennan Hyden, Gerald A Tuskan, Paul E Abraham, Xiaohan Yang

**Affiliations:** Biosciences Division, Oak Ridge National Laboratory, Oak Ridge, TN 37831, USA; Biosciences Division, Oak Ridge National Laboratory, Oak Ridge, TN 37831, USA; The Center for Bioenergy Innovation, Oak Ridge National Laboratory, Oak Ridge, TN 37831, USA; Biosciences Division, Oak Ridge National Laboratory, Oak Ridge, TN 37831, USA; Biosciences Division, Oak Ridge National Laboratory, Oak Ridge, TN 37831, USA; The Center for Bioenergy Innovation, Oak Ridge National Laboratory, Oak Ridge, TN 37831, USA; Biosciences Division, Oak Ridge National Laboratory, Oak Ridge, TN 37831, USA; The Center for Bioenergy Innovation, Oak Ridge National Laboratory, Oak Ridge, TN 37831, USA; Biosciences Division, Oak Ridge National Laboratory, Oak Ridge, TN 37831, USA; The Center for Bioenergy Innovation, Oak Ridge National Laboratory, Oak Ridge, TN 37831, USA

## Abstract

Anti-CRISPR proteins are very efficient for inhibiting CRISPR/Cas9-based genome editing tools in both herbaceous and woody plant species.

Dear Editor,

Clustered regularly interspaced short palindromic repeats/CRISPR associated protein (CRISPR/Cas) systems have revolutionized genome engineering in plants (Hassan et al. 2021) via specific control of genetic modifications and transcriptional activities (Liu et al. 2018; Wolter et al. 2018; Pan et al. 2022). However, knockout or overexpression of many critical genes may cause pleiotropic effects, which could be limited by conditional and tissue-specific gene modifications (Pfeiffer et al. 2022). Hence, it is necessary to develop spatially and temporally controllable CRISPR/Cas-based tools for precise genome engineering (Hoffmann et al. 2019; Calvache et al. 2022). Anti-CRISPR (Acr) proteins, natural inhibitors for CRISPR/Cas systems, have utility in biodesign strategies aimed at regulating Cas activities (Marino et al. 2020). Acr proteins inhibit CRISPR/Cas activities by either blocking (i) DNA-binding activity or (ii) DNA cleavage activity of Cas proteins (Marino et al. 2020). Multiple Acr proteins have been tested in mammalian cells and yeast (*Saccharomyces cerevisiae*), including AcrIIA4 (inhibits SpCas9), AcrVA1 (inhibits Cas12a), and AcrIIA5 (potentially inhibits all Cas9 orthologs) (Marino et al. 2020; Zhang and Marchisio 2022). Acr proteins can potentially regulate Cas activity at the post-translational level (Calvache et al. 2022). For example, AcrIIA4 has been used to limit CRISPR/Cas activity to particular environments (e.g. blue-light) in nonplant cells (Bubeck et al. 2018; Zhang and Marchisio 2021). Also, cell-specific genome editing mediated by CRISPR/Cas9 was achieved through microRNA-dependent expression of Acr proteins in human cells (Hoffmann et al. 2019). However, Acrs have not been widely used for tunable genome editing in plants. So far, only AcrIIA4 and AcrVA1 have been evaluated in a single plant species (*Nicotiana benthamiana*) based on transient expression through leaf infiltration and viral delivery (Calvache et al. 2022). AcrIIA5 activity remains to be evaluated in plants, and performance differences between transient and stable expression of Acrs remain unanswered. Therefore, we evaluated the performance of AcrIIA4 and AcrIIA5 activities in herbaceous and woody plant species using both transient expression and stable transformation approaches. We tested the effects of AcrIIA4 and AcrIIA5 activities on the SpCas9-based adenine base editor (ABE7) in the herbaceous plants Arabidopsis (*Arabidopsis thaliana*) and *N. benthamiana*, and the woody plant hybrid poplar “717” (*Populus tremula* × *P. alba* hybrid clone INRA 717-1B4), using both leaf-infiltration and protoplast-based transient expression. The activity of AcrIIA4 on ABE7 was further investigated in Arabidopsis via *Agrobacterium tumefaciens*-mediated stable genetic transformation.

Transient expression of Acr activity was evaluated in *N. benthamiana*, *A. thaliana*, and hybrid poplar “717” using a 35S promoter and codon optimized AcrIIA4 or AcrIIA5 (Supplemental Fig. S1). Previously generated biosensor systems (BS2s), containing a mutated nonfunctional GFP gene and a single gRNA targeting the mutated region, capable of measuring ABE7 activity (Yuan et al. 2021) were used to evaluate Acr activity (Supplemental Table S1), whereby restoration of GFP signals indicates active and successful base editing by ABE7 (Li et al. 2018; Yuan et al. 2021). In *A. thaliana* and hybrid poplar “717”, constructs were introduced to protoplast cells via PEG-mediated transformation; in *N. benthamiana*, constructs were delivered by agrobacterium-mediated leaf infiltration (Supplemental Methods S1). Positive GFP signal was detected in Arabidopsis and poplar protoplast cells as well as in *N. benthamiana* leaves when ABE7 and BS2s were co-expressed transiently (Fig. 1, A and B; Supplemental Figs. S2 and S3). Introducing Acr expression constructs negatively impacted ABE7 activity and resulted in cells lacking GFP signal, which is similar to the BS2s only treatment (Fig. 1, A and B; Supplemental Figs. S2 and S3). As a control, a luciferase expression plasmid was co-delivered with ABE7 and BS2s to eliminate the possibility that cells lacking GFP signal might be caused by low transformation efficiency. Three independent experiments were conducted to validate the inhibition activity of Acrs and similar results were obtained each time, indicating AcrIIA4 and AcrIIA5 can block the ABE7 activity in plants. The editing efficiency of ABE was assessed to quantitively measure the inhibition effect of Acrs. The number of cells with positive GFP signals were calculated based on 4 independent fields from the samples with the highest transformation efficiency. For the target site of BS2-1, the average editing efficiency of ABE7 was 50%, whereas 33% editing efficiency was observed for the target site BS2-2 in Arabidopsis protoplast cells (Fig. 1C). For both target sites, zero GFP positive cells were obtained when ABE7 was transformed with either AcrIIA4 or AcrIIA5 (Fig. 1C), suggesting that the editing activity of ABE7 was substantially abolished independent of target sites. Sporadic GFP-expressing cells were also detected in one batch of experiment, possibly due to failure of co-transformation of the AcrIIA4 or AcrIIA5 construct (Supplemental Fig. S4). Similar results were obtained in the poplar protoplast assay (Fig. 1, A and D; Supplemental Fig. S2), suggesting both AcrIIA4 and AcrIIA5 have substantial inhibition activity for the base editor ABE7 in both herbaceous and woody plant species. These results are consistent with previous studies in yeast and mammalian cells (Liang et al. 2020; Zhang and Marchisio 2022). Moreover, our results demonstrate that the BS2s allow us to test Acrs activity efficiently in plants. Currently, multiple synthetic Acr small molecules have been identified from a high-throughput platform in human cells for dose and temporal control of SpCas9 (Maji et al. 2019). The platform we describe here offers a high-throughput approach for identifying additional control elements of CRISPR/Cas9 in plants.

**Figure 1.**
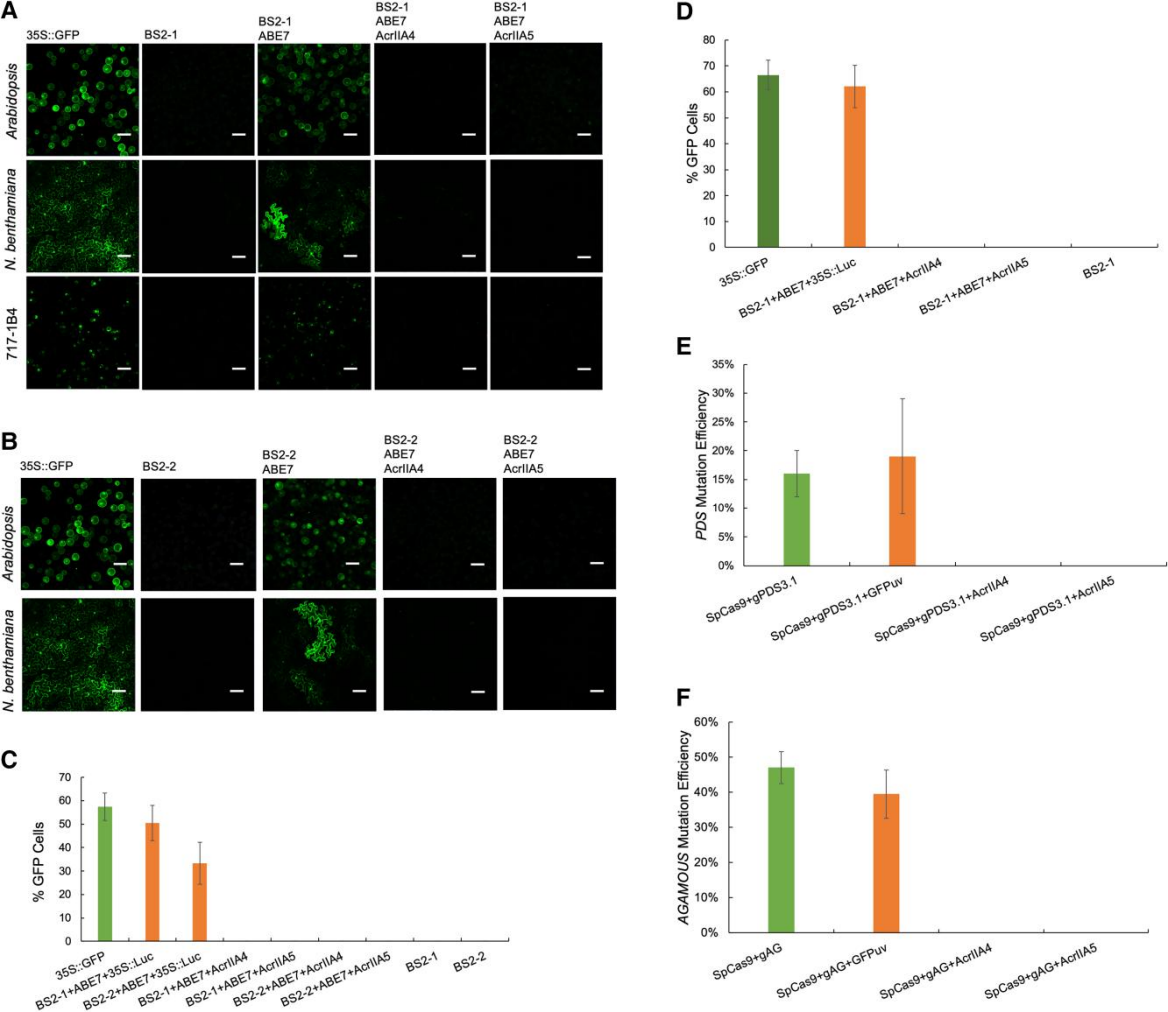
Investigation of Acrs’ inhibition activities for CRISPR/Cas9-based genome editing tools via transient assays. Same positive control images of 35s::GFP were used in A) and B). A) Evaluation of the AcrIIA4 and AcrIIA5 inhibition activities for SpCas9-based base editor ABE7 by BS2-1 through Arabidopsis and poplar protoplast transformation and *N. benthamiana* leaf infiltration. Scale bar = 100 μm. B) Evaluation of the AcrIIA4 and AcrIIA5 inhibition activities for SpCas9-based base editor ABE7 by BS2-2 through Arabidopsis protoplast transformation and *N. benthamiana* leaf infiltration. Scale bar = 100 μm. C) Analysis of GFP-positive cells with and without the Acr proteins in Arabidopsis protoplast. Error bars indicate standard deviation (SD). Sample size is 4. D) Analysis of GFP-positive cells with and without the Acr proteins in a *Populus* hybrid (*Populus tremula* × *P. alba* hybrid clone INRA 717-1B4) protoplast. Error bars indicate SD. Sample size is 4. E) Editing efficiency of SpCas9 targeting the *NbPDS* in the absence and in the presence of AcrIIA4 and AcrIIA5. Error bars indicate SD. Sample size ranges from 3 to 6. F) Editing efficiency of SpCas9 targeting the *NbAGAMOUS* in the absence and in the presence of AcrIIA4 and AcrIIA5. Error bars indicate SD. Sample size ranges from 4 to 6.

To further evaluate the inhibition activity of Acrs to SpCas9/sgRNA directed target mutagenesis in plants, we tested the ability of AcrIIA4 and AcrIIA5 to prevent SpCas9-induced mutagenesis in *N. benthamiana* for 2 previously well-characterized targets, *phytoene desaturase* (PDS) and *AGAMOUS* (AG) (Supplemental Table S1;Ellison et al. 2021). The average efficiency for SpCas9 editing was 19% for PDS and 40% for AG at 10 d post-infiltration and transient expression of the SpCas9/sgRNA construct (Fig. 1, E and F; Supplemental Table S2). Co-infiltration of a GFPuv construct with the SpCas9/sgRNA system resulted in a similar average editing efficiency of 16% for PDS and 47% for AG (Fig. 1, E and F; Supplemental Table S2). Introducing either the AcrIIA4 or AcrIIA5 expression construct with co-infiltration reduced SpCas9 editing efficiency to undetectable levels for both target genes (Fig. 1, E and F; Supplemental Table S2), suggesting that both AcrIIA4 and AcrIIA5 can prevent SpCas9/sgRNA-mediated target mutagenesis in the genome of *N. benthamiana*. These results are consistent with a previous report in mammalian cells (Garcia et al. 2019), suggesting AcrIIA5 inhibits genome editing with comparable potency to AcrIIA4 in plants.

To test the Acrs activity following stable genetic transformation, the transcriptional units of AcrIIA4-BS2 and ABE7 were integrated into the Arabidopsis genome together via 2 independent constructs (Supplemental Methods S1). The expression cassette of AcrIIA4 was integrated into the same construct of BS2-1 to make the AcrIIA4-BS2 construct (Fig. 2A). Eleven stable transgenic plants were generated by *Agrobacterium*-mediated co-transformation of the ABE7 and AcrIIA4-BS2 constructs (Supplemental Fig. S5). Among them, 4 transgenic plants with high expression level of AcrIIA4 did not generate GFP signals, whereas 6 transgenic plants with lower expression level of AcrIIA4 could still generate GFP signals, suggesting that the inhibition activity of AcrIIA4 depends on its concentration (Fig. 2, B and C). All transgenic plants co-transformed with BS2 and ABE7 showed strong GFP signals (Fig. 2B). These results demonstrate that AcrIIA4 inhibits SpCas9 activity in a dose-dependent manner when integrated in the plant genome, which is consistent with the previous transient assay in *N. benthamiana* (Calvache et al. 2022), and thus lends support for using AcrIIA4 in biodesign strategies to regulate SpCas9-based genome engineering tools in plants.

**Figure 2.**
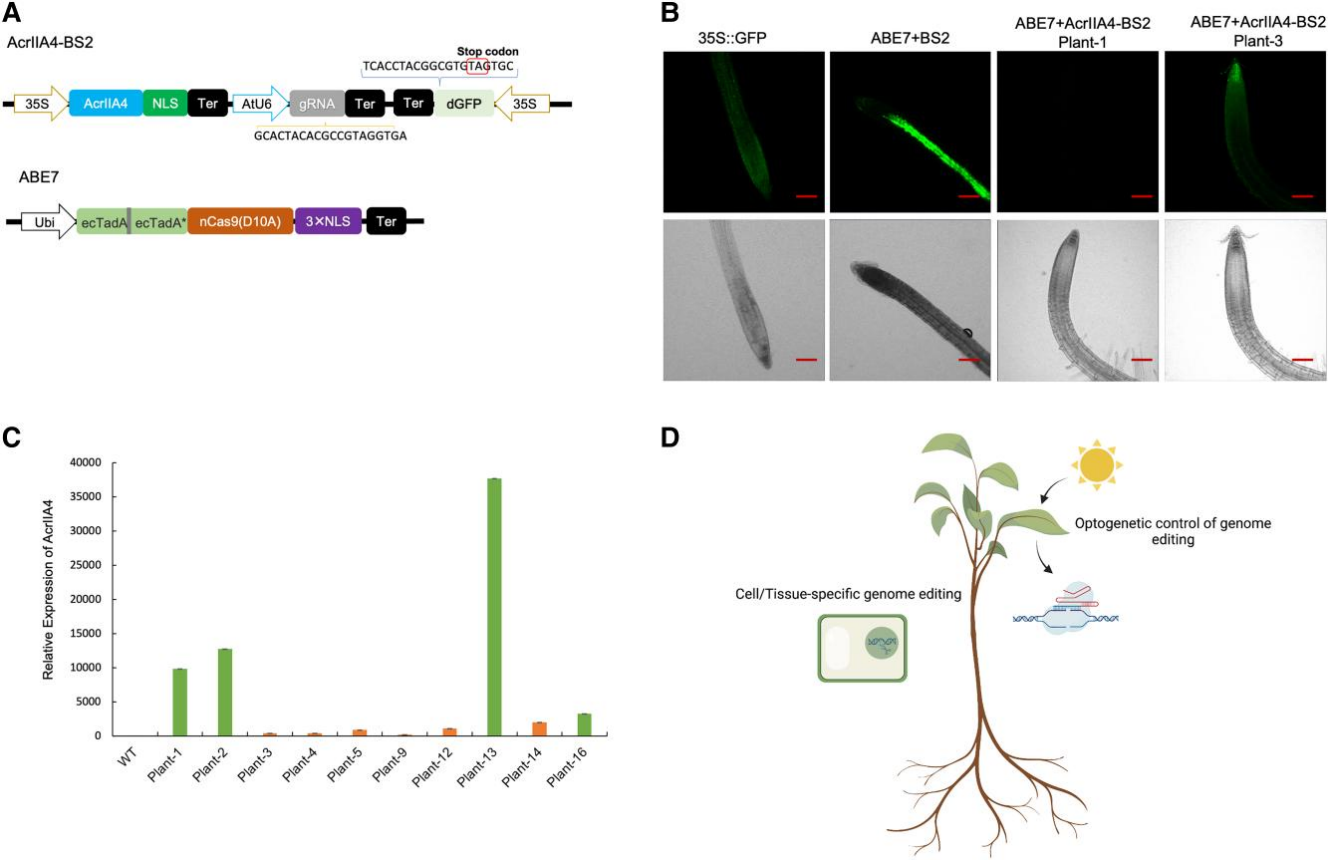
Investigation of AcrIIA4 inhibition activities for ABE7 via stable transformation. **A)** Construct design for AcrIIA4-BS2 and ABE7 (Li et al. 2018). NLS indicates nuclear localization signal. Ter indicates terminator. Ubi represents maize *Ubiquitin-1* promoter. **B)** Root pictures of representative transgenic Arabidopsis plants. The upper panel pictures were taken under fluorescence and lower panel pictures were taken under bright light. Scale bar = 100 μm. **C)** Relative expression level of AcrIIA4 in 10 transgenic plants. Error bars indicate SD. Sample size is 3. **D)** Potential applications of Acrs in plant tunable genome engineering.

In summary, we demonstrate that Acr proteins are effective in inhibiting CRISPR/Cas9-based genome editing tools in both herbaceous and woody plant species. As such, previously described biosensors for CRISPR-based tools (Yuan et al. 2021) proved useful in their utility for screening genome editing activity or the lack thereof. We demonstrate the Acr-mediated inhibition of ABE7 in transgenic plants. Comparable with earlier observations (Calvache et al. 2022), the inhibitory effect of AcrIIA4 is dose-dependent and thus can be exploited by future biodesign efforts aimed at fine-tuning the activities of CRISPR/SpCas9 systems. Overall, these results lay the foundation for future application of Acr proteins in plant synthetic biology. Opportunities for the application of Acrs in tunable genome editing in plants include (i) integration of cell-specific miRNA binding sites to Acrs (Hoffmann et al. 2019) to build cell type-specific Cas9-ON switch for cell type-specific plant genome editing and (ii) fusion of a light-responsible domain with Acrs to enable optogenetic control of CRISPR/Cas system for inducible plant genome editing (Bubeck et al. 2018), as illustrated in Fig. 2D.

## Supplementary Material

kiad076_Supplementary_Data

## Data Availability

The plasmids generated in this study will be available at Addgene.

## Supplemental data

The following materials are available in the online version of this article.

**Supplemental Figure S1.** Construct design for AcrIIA4 and AcrIIA5 expression.

**Supplemental Figure S2.** Bright field of pictures in Fig. 1A.

**Supplemental Figure S3.** Bright field of pictures in Fig. 1B.

**Supplemental Figure S4.** Evaluation of the AcrIIA4 and AcrIIA5 inhibition activities for SpCas9-based base editor ABE7 by BS2s through Arabidopsis protoplast transformation.

**Supplemental Figure S5.** Transgenic plants confirmation by PCR.

**Supplemental Table S1.** Targeted genes and gRNA sequences of the editing experiments.

**Supplemental Table S2.** Summary of Synthego's ICE results for the SpCas9-mediated editing assay.

**Supplemental Table S3.** Plasmids used and constructed in this study.

**Supplemental Table S4.** Primers used in this study.

**Supplemental Dataset S1.** Annotated vector sequences.
