# Supplementary material for: Expanding the application of anti-CRISPR proteins in plants for tunable genome editing
Source: Plant Physiol. 2023 Feb 9;192(1):60–4. doi: 10.1093/plphys/kiad076 (PMC10152675; doi:10.1093/plphys/kiad076)
Supplement: kiad076_Supplementary_Data [file kiad076_supplementary_data.zip › Supplemental Data.pdf]

Supplemental Figure S1.

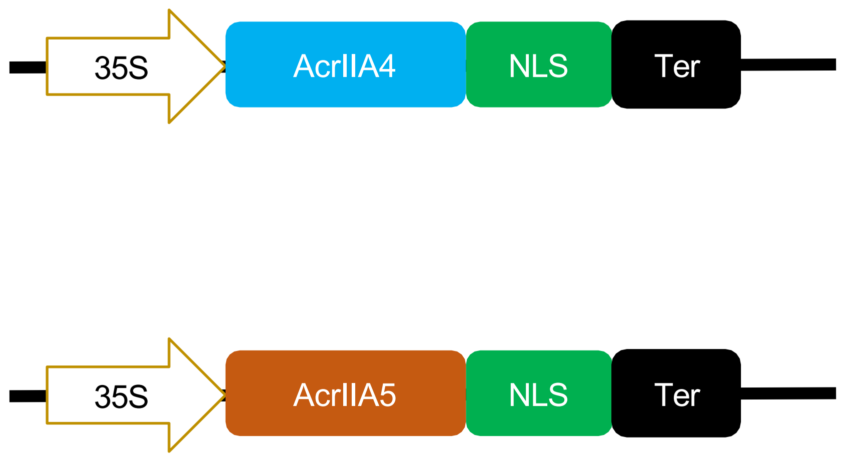

Supplemental Figure S1. Construct design for AcrIIA4 and AcrIIA5 expression. 35S represents the 35S promoter, NLS represents the nucleoplasmin nuclear localization signal, and Ter represents the NOS terminator

Supplemental Figure S2.

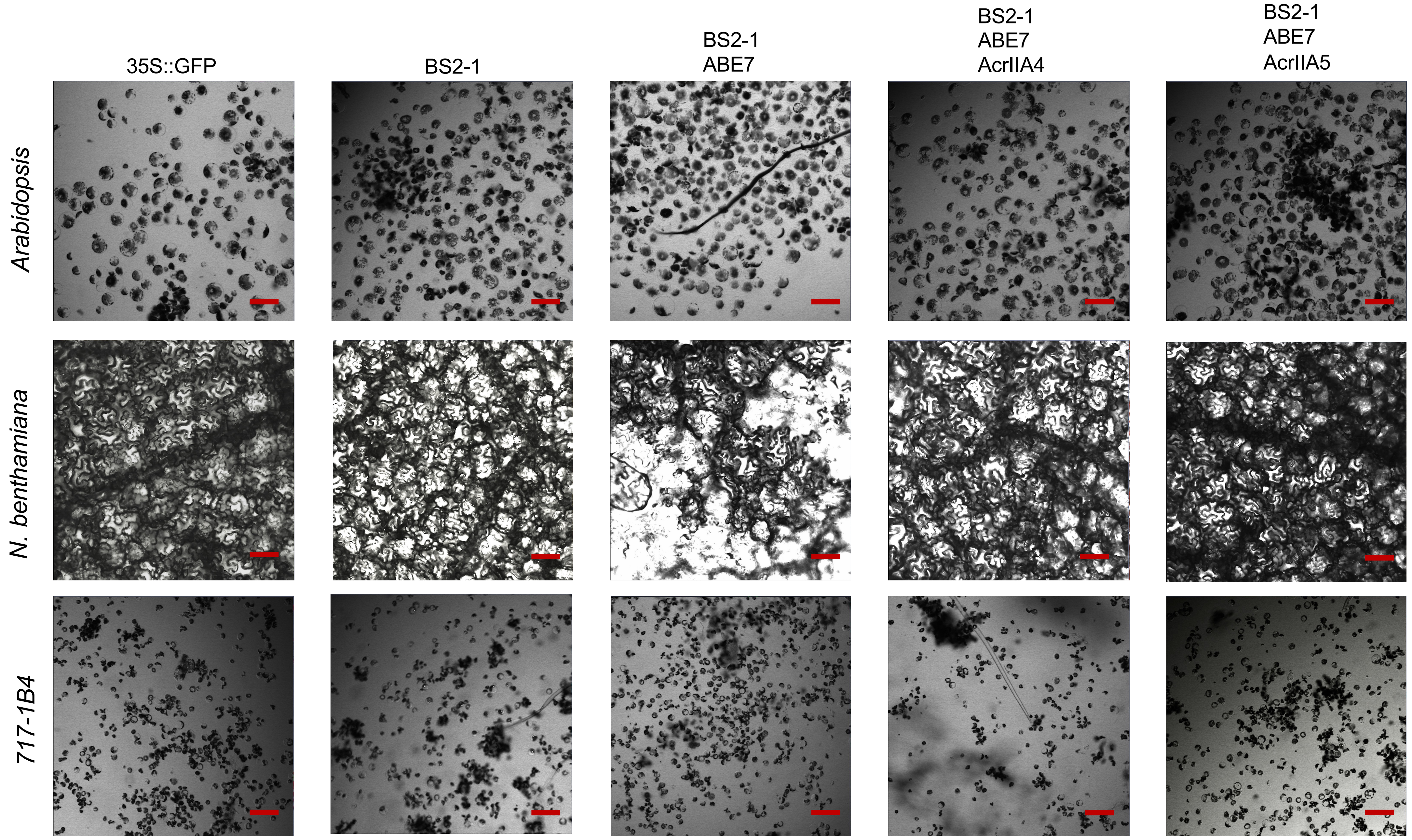

Supplemental Figure 2. Bright field of pictures in Figure 1A. The pictures were taken with a Zeiss LSM 710 confocal microscope under bright light. Scale bar = 100 μm.

Supplemental Figure S3.

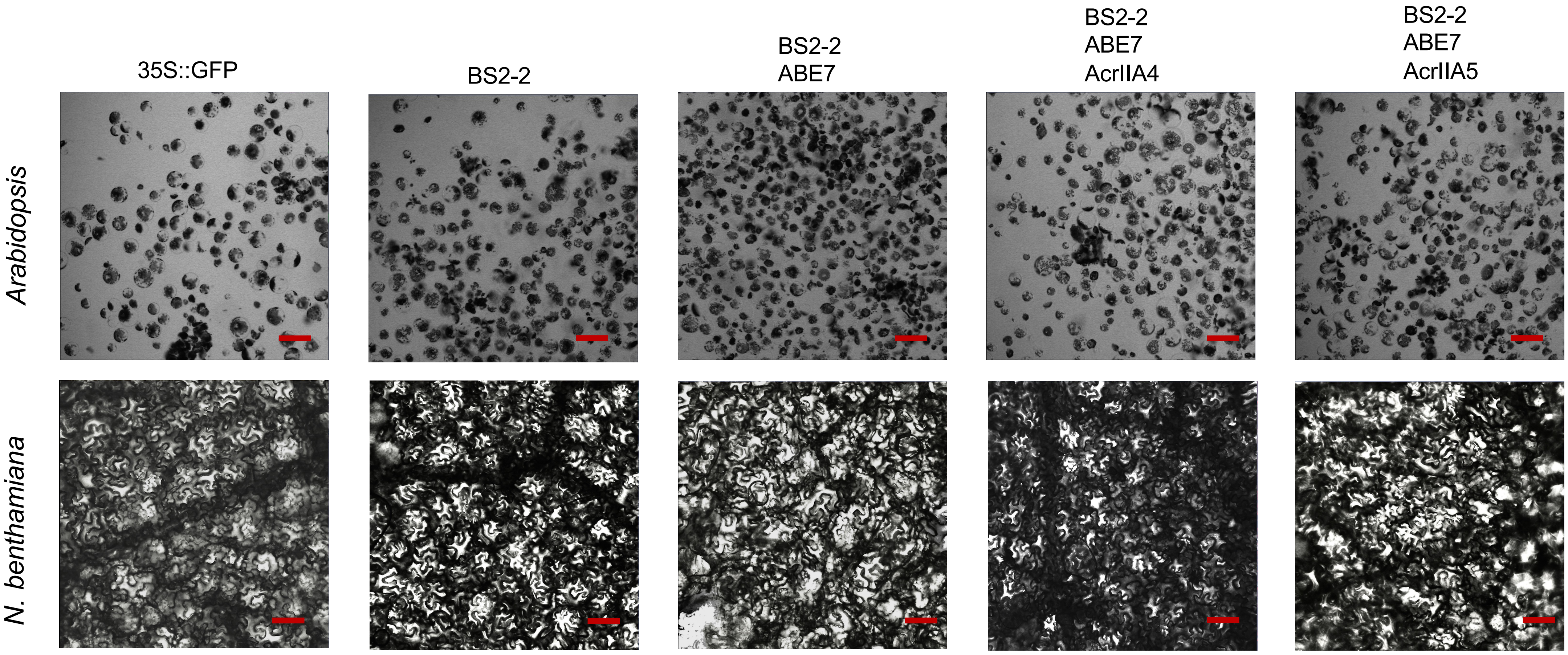

Supplemental Figure S3. Bright field of pictures in Figure 1B. The pictures were taken with a Zeiss LSM 710 confocal microscope under bright light. Scale bar = 100 μm.

Supplemental Figure S4.

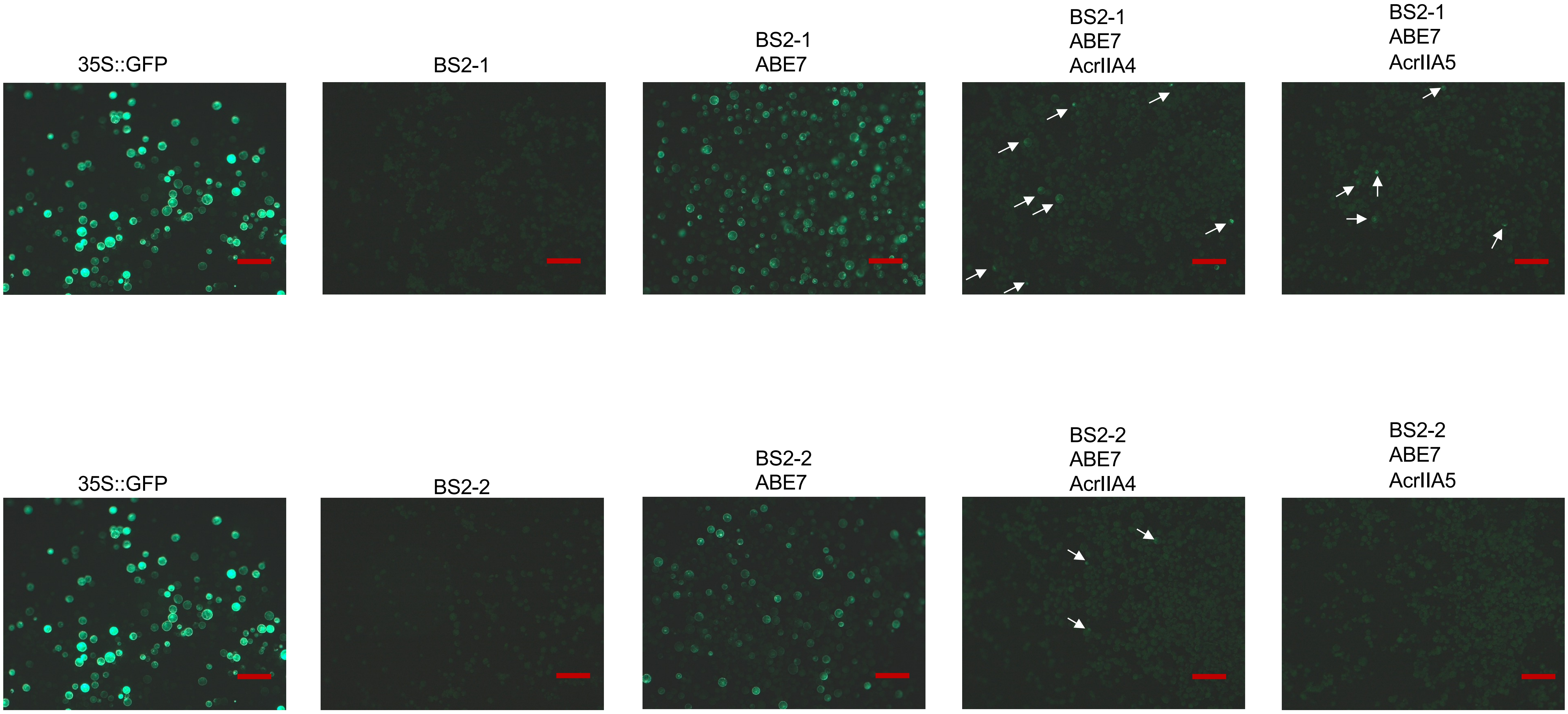

Supplemental Figure S4. Evaluation of the AcrIIA4 and AcrIIA5 inhibition activities for SpCas9-based base editor ABE7 by BS2s through *Arabidopsis* protoplast transformation. Scale bar = 100 µm. Pictures were taken with a Zeiss Imager.A2 microscope under fluorescence. White arrow indicates the GFP-expressing cells in samples co-transformed with BS2s, ABE7, and anti-CRISPR proteins. Same positive control images of 35s::GFP were used in upper and lower panel.

Supplemental Figure S5.

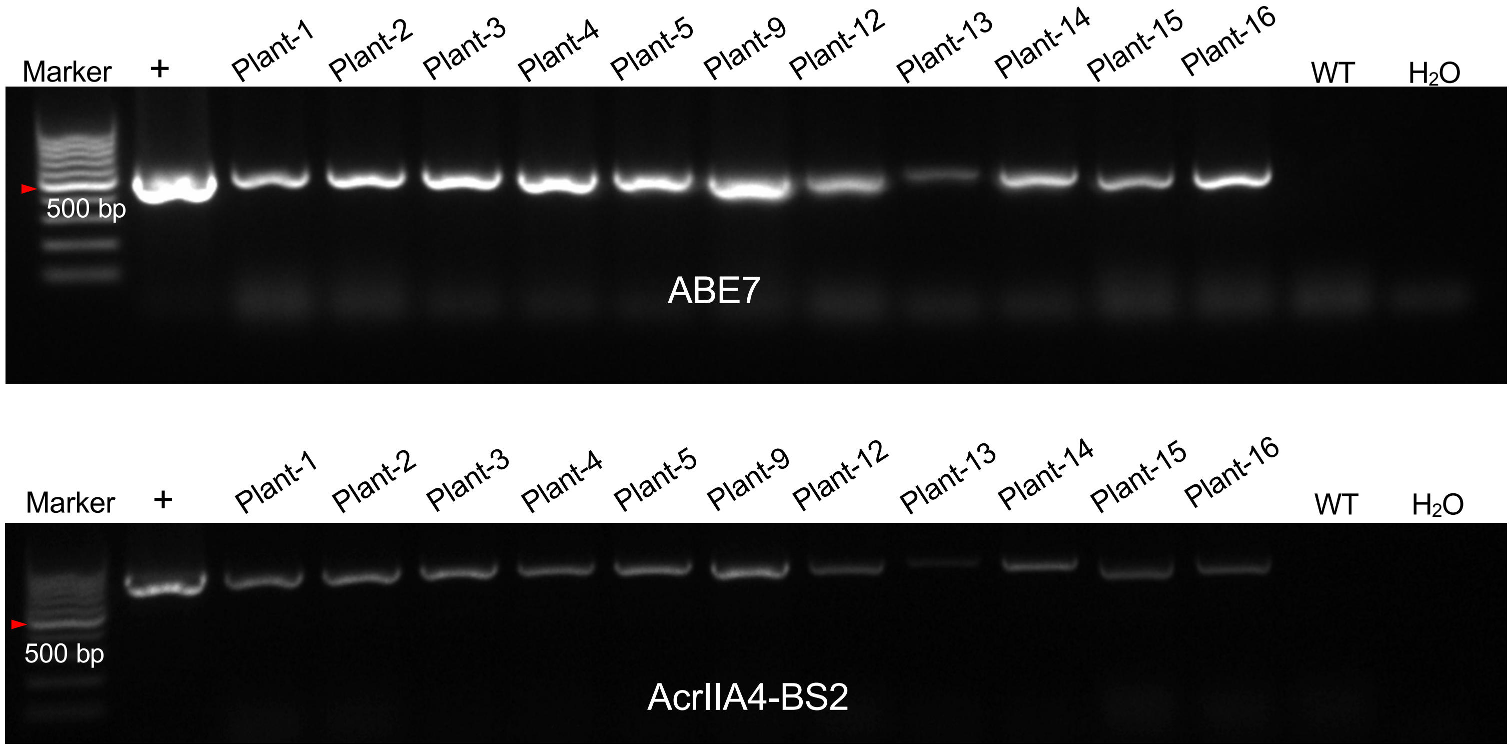

Supplemental Figure S5. Transgenic plants confirmation by PCR

Supplemental Table S1. Targeted genes and gRNA sequences of the editing experiments.

Target gene	gRNA name	Protospacer sequence
BS2-1	gdGFP2a	GCACTACACGCCGTAGGTGA
BS2-2	gdGFP2b	CGTGCTACTTCATGTGGTCG
Niben101Scf12205g00011.1 (*NbAGAMOUS*)	gAG	GTGTGAAAGAAACAATTGAG
Niben101Scf14708g00023.1 (*NbPDS*)	gPDS3.1	TTGGTAGTAGCGACTCCATG

Supplemental Table S2. Summary of Synthego's ICE results for the SpCas9-mediated editing assay

Treatment	Replicate	ICE	KO-Score	R^2
**SpCas9/gPDS3.1**	**Sample1**	**21**	**21**	**0.97**
	**Sample 2**	**11**	**11**	**0.96**
	**Sample 3**	**16**	**16**	**0.97**
**SpCas9/gPDS3.1+GFPuv**	**Sample 3**	**8**	**8**	**0.97**
	**Sample 4**	**15**	**15**	**0.95**
	**Sample 5**	**34**	**34**	**0.95**
**SpCas9/gPDS3.1+AcrIIA4**	**Sample 2**	**0**	**0**	**1**
	**Sample 3**	**0**	**0**	**1**
	**Sample 4**	**0**	**0**	**1**
	**Sample 5**	**0**	**0**	**1**
	**Sample 6**	**0**	**0**	**1**
**SpCas9/gPDS3.1+AcrIIA5**	**Sample 1**	**0**	**0**	**1**
	**Sample 2**	**0**	**0**	**1**
	**Sample 3**	**0**	**0**	**1**
	**Sample 4**	**0**	**0**	**1**
	**Sample 5**	**0**	**0**	**1**
	**Sample 6**	**0**	**0**	**1**
**SpCas9/gAG**	**Sample 1**	**48**	**42**	**0.94**
	**Sample 2**	**43**	**38**	**0.95**
	**Sample 5**	**43**	**37**	**0.96**
	**Sample 6**	**54**	**45**	**0.93**
**SpCas9/gAG+GFPuv**	**Sample 1**	**29**	**29**	**0.95**
	**Sample 3**	**44**	**41**	**0.92**
	**Sample 5**	**38**	**36**	**0.91**

	**Sample 6**	**47**	**47**	**0.92**
**SpCas9/gAG+AcrIIA4**	**Sample 1**	**0**	**0**	**1**
	**Sample 2**	**0**	**0**	**1**
	**Sample 4**	**0**	**0**	**1**
	**Sample 5**	**0**	**0**	**1**
	**Sample 6**	**0**	**0**	**1**
**SpCas9/gAG+AcrIIA5**	**Sample 1**	**0**	**0**	**1**
	**Sample 2**	**0**	**0**	**1**
	**Sample 3**	**0**	**0**	**1**
	**Sample 4**	**0**	**0**	**1**

Supplemental Table S3. Plasmids used and constructed in this study.

**Name**	**Description**	**References**
BS2-1	A dark-GFP mutant (gfp2a, Q69 > TAG) with corresponding sgRNA	(Yuan et al. 2021a)
BS2-2	A dark-GFP mutant (gfp2b, Q80 > TAG) with corresponding sgRNA	(Yuan et al. 2021a)
ABE7	Base editor expression vector	(Li et al. 2018)
pYL_ORNL_001	AcrIIA4 expression vector	This study
pYL_ORNL_003	AcrIIA5 expression vector	This study
pYL_ORNL_004	Gene editing vector for *NbPDS*	This study
pYL_ORNL_005	Gene editing vector for *NbAG*	This study
pGFPuv	*eYGFPuv* expression vector	(Yuan et al. 2021b)
pKSE401-U3	SpCas9/gRNA expression vector	(Yuan et al. 2022)
AcrIIA4-BS2	AcrIIA4 expression cassette integrated into BS-1 vector	This study
pMOD_A5801	Luciferase expression vector	(Maher et al. 2020)
pGFPGUSPlus	GFP expression vector	(Vickers et al. 2007)

Supplemental Table S4. Primers used in this study.

Primer name	Primer sequence (5′-3′)
oYL082_*AcrIIA4*_qRT_F	GGACTACACCGTCAAGCTCTC
oYL083_*AcrIIA4*_qRT_R	CGGATTCACTGATGACGTACTC
At*Act2*_qRT_F	GTCGTACAACCGGTATTGTGCTG
At*Act2*_qRT_R	GGTAATCAGTAAGGTCACGTCCAGC
ABE7_F	GAAAAATGGATTCATAGCTTCTAATTGCTACCTGCAGGAGATTT
ABE7_R	GCGCAATGAGATTCCCG
oYL029_BS2_F	CAGGAAACAGCTATGAC
oGY135_BS2_R	GACGACGGCAACTACAAGAC
*AG*_genotyping_F	ACACTCTTTCCCTACACGACGCTCTTCCGATCTAAAGACGGCCAT GAGAATATAATGAATC
*AG*_genotyping_R	GACTGGAGTTCAGACGTGTGCTCTTCCGATCTGTGGATATGAGA ACCACCCTATTCTG
*PDS*_genotyping_F	TAATCTTGGTTTGTAATCCACAACG
*PDS*_genotyping_R	ACGAAATGATGATGATGATAACGC
PDS_Sequencing	GCCGTTAATTTGAGAGTCCAAG
oGY1219_*AcrIIA4*_F	TAATCTGGGGACCTGCAGGCATGCAAGCTTCAGAAGACCAAAG GGCAAT
oGY1219_*AcrIIA4*_R	CGTTGTAAAACGACGGCCAGTGCCACCATGATTACGAATTCCCG ATC

References:

Li C, Zong Y, Wang YP, Jin S, Zhang DB, Song QN, Zhang R, Gao CX. 2018. Expanded base editing in rice and wheat using a Cas9-adenosine deaminase fusion. *Genome Biology* **19**: 1-9.

Maher MF, Nasti RA, Vollbrecht M, Starker CG, Clark MD, Voytas DF. 2020. Plant gene editing through de novo induction of meristems. *Nature Biotechnology* **38**: 84-89.

Vickers CE, Schenk PM, Li D, Mullineaux PM, Gresshoff PM. 2007. pGFPGUSPlus, a new binary vector for gene expression studies and optimising transformation systems in plants. *Biotechnol Lett* **29**: 1793-1796.

Yuan G, Martin S, Hassan MM, Tuskan GA, Yang X. 2022. PARA: A New Platform for the Rapid Assembly of gRNA Arrays for Multiplexed CRISPR Technologies. *Cells* **11**: 2467.

Yuan GL, Hassan MM, Yao T, Lu HW, Vergara MM, Labbe JL, Muchero W, Pan CT, Chen JG, Tuskan GA et al. 2021a. Plant-Based Biosensors for Detecting CRISPR-Mediated Genome Engineering. *Acs Synth Biol* **10**: 3600-3603.

Yuan GL, Lu HW, Tang D, Hassan MM, Li Y, Chen JG, Tuskan GA, Yang XH. 2021b. Expanding the application of a UV-visible reporter for transient gene expression and stable transformation in plants. *Hortic Res-England* **8**: 234.

Supplemental Methods S1

Plant materials

Arabidopsis (*Arabidopsis thaliana*) wild-type Col-0 plants and *Nicotiana benthamiana* (GenBank: PRJNA170566) plants were grown in soil within the growth chamber with 12 light/ 12 h dark period with light intensity of 100 μmol m^{-2} s^{-1} at 21 °C. The *in vitro* grown *P. tremula* x *P. alba* '717-1B4' were maintained in MS medium in a growth room with 16 h light/8 h dark period with light intensity of 100 μmol m^{-2} s^{-1} at 23 °C. The plants were sub-cultured into a fresh medium monthly.

Plasmids construction

All constructs used or created in this study are listed in Supplemental Table S3, and annotated vector sequences (GenBank files) are provided in supplemental data S1. All plasmids created in this study were constructed using Gibson assembly with NEBuilder® HiFi DNA Assembly master mix (NEB, Cat. No. E2621) or Golden Gate assembly using the NEBridge® Golden Gate Assembly Kit (BsaI-HF® v2) (NEB, Cat. No. E1602). The vectors BS2-1, BS2-2, pKSE401-U3, and pGFPuv have been previously reported by us (Yuan et al. 2021a; Yuan et al. 2021b; Yuan et al. 2022a). The base editor expression vector, ABE7 (Addgene no.115621, https://www.addgene.org/115621/), GFP expression vector, pGFPGUSPlus (Addgene no.64401, https://www.addgene.org/64401/), and luciferase expression vector, pMOD_A5801 (Addgene no. 127223, https://www.addgene.org/127223/) were ordered from Addgene. Amino acid sequences of AcrIIA4 and AcrIIA5 were obtained from previous reports (Hynes et al. 2017; Song et al. 2019) and codon optimized for *Nicotiana benthamiana* using IDT Codon Optimization Tool (https://www.idtdna.com/CodonOpt). To build the pYL_ORNL_001 and pYL_ORNL_003, the codon optimized AcrIIA4 and AcrIIA5 fused with the nucleoplasmin nucleus localization signal were chemically synthesized by Integrated DNA Technology (Coralville, IA) and inserted into XbaI (NEB, Cat. No. R0145) and SacI (NEB, Cat. No. R3156) digested pGFPGUSPlus binary vector for plant expression. To make the pYL_ORNL_004 and pYL_ORNL_005, chemically synthesized gRNAs fragments were inserted into the pKSE401-U3 plasmid with Golden Gate assembly. To make the AcrIIA4-BS2 construct, the AcrIIA4 expression cassette was amplified by polymerase chain reaction (PCR) using Q5 Hot Start High-Fidelity DNA Polymerase (NEB, Cat. No. M0492) and inserted into the BS2-1 vector digested by HindIII (NEB, Cat. No. R3104). The sequences of all plasmids were confirmed by Sanger sequencing. Primers for amplification and sequencing are listed in Supplemental Table S4.

***Nicotiana benthamiana* leaf infiltration**

Five to six weeks old *N. benthamiana* plants were used. The *Agrobacterium tumefaciens* strain GV3101 harboring the plasmid of interest was infiltrated into the leaves of *N. benthamiana* using a 1mL syringe without a needle as described previously (Li 2011; Yuan et al. 2022b). Briefly, cultures containing the vector of interest were grown in lysogeny broth (LB) medium for 36 ~ 48 hours and resuspended in agroinfiltration buffer (10 mM MES, pH 5.6, 10 mM $MgCl_2$ and 200 μM acetosyringone) to an optical density of 0.5 at 600 nm (OD600). Bacterial suspensions were incubated for 2 to 4 hours at room temperature and then mixed for co-expression

experiments. Agroinfiltrations were carried out through the abaxial surface of the three youngest fully expanded leaves of each plant with a 1-ml needle-free syringe. Each treatment includes six replicates.

Protoplast transformation

The isolation and transient transformation of *Arabidopsis* and poplar leaf mesophyll protoplasts were performed as described previously (Xie et al. 2018; Yuan et al. 2022b). In brief, full expanded leaves from one-month-old *Arabidopsis* and *P. tremula* x *P. alba* '717-1B4'plants were sliced into strips and immersed into enzyme solution (0.4 M mannitol, 20 mM KCl, 20 mM MES, 10 mM $CaCl_2$, 5 mM β-mercaptoethanol, 0.1% (W/V) BSA, 0.8% (W/V) macerozyme R10, and 3% (W/V) cellulase R10). After 3-5 hours, the protoplasts were washed with W5 solution (154 mM NaCl, 125 mM $CaCl_2$, 5 mM KCl, and 2 mM MES).

5ug plasmids of each construct were transfected into protoplasts using PEG/Ca^{2+} solution (100 mM $CaCl_2$, 0.2 M mannitol, 40% (W/V) PEG4000). After incubating in W5 solution for 24-48 hours, the protoplasts were harvested for GFP visualization. Three independent experiments were conducted for each treatment.

Stable transformation in *Arabidopsis*

The *Agrobacterium* strain 'GV3101' was used for the transformation of *Arabidopsis* wild type 'Col-0' via the floral dip method with modification as described previously (Yuan et al. 2021b). T1 seeds co-transformed with two constructs were selected on K1 medium with 50 mg/L Kanamycin and 25 mg/L Hygromycin. Seeds were put in 4 ^{o}C under dark for 2 days and then germinated for 6 to 8 days under 100 to 150 μmol m^{-2} s^{-1} fluorescent warm white light, 12 h of light/12 h of dark, 20°C, and 70% humidity. Then, seedlings were collected for GFP visualization. Seedlings were transferred to soil after GFP visualization and cultivated in controlled-climate chambers with conditions described before. DNA samples were collected from 4-5 weeks old plants using a modified SDS protocol (Ahmed et al. 2009). The DNA was used as template for PCR amplification of the transgenes with primers listed on Supplemental Table S4 and using OneTaq® Hot Start Quick-Load® 2X Master Mix (NEB, Cat. No. M0488).

Microscopy analysis

The fluorescence of GFP was visualized and imaged using a Zeiss LSM 710 confocal microscope with a FITC (green) filter. The EGFP dye was excited within the spectral range of 493 to 584 nm.

Determination of Cas9-mediated editing efficiency

For determination of editing efficiencies on plants transiently expressing anti-CRISPR proteins and their corresponding controls, approximately 150 mg of leaf tissue was collected from each of the six replicates at 10 dpi. Genomic DNA was extracted from leaf tissue following a modified SDS protocol (Ahmed et al. 2009). The DNA was used as template for PCR amplification of the targeted sites with primers listed on Supplemental Table S4 and using GoTaq® Master Mixes

(Promega, Cat. No. M7122). Subsequently, PCR products were analyzed in 1% (W/V) agarose gel electrophoresis, purified with the PCR Product Cleanup Reagent (ZYMO RESEARCH, Cat. No. D4030) following manufacturer instructions and Sanger-sequenced. Finally, sequencing results were analyzed using Synthego CRISPR Performance Analysis (https://ice.synthego.com/#/) to determine the editing efficiency.

RNA isolation and RT-qPCR

Leaf samples from 4-5 weeks old transgenic plants were used for total RNA isolation using the GenElute™ Total RNA Purification Kit (Sigma-Aldrich, Cat. No. RNB100). Total RNA was treated with TURBO DNA-free Kit (Invitrogen, Cat. No. AM1907) following manufacturer´s instructions. Aliquots of 500 ng of the treated RNA were used for cDNA synthesis and determine the expression levels for each gene in triplicated 12 μl reactions with the Luna Universal One-Step RT-qPCR Kit (NEB, Cat. No. E3005) using the Bio-Rad CFX96 Real-Time system. Relative expression of AcrIIA4 for each sample were calculated by ΔΔCT method (Schmittgen and Livak 2008). Primers used for RT-qPCR reactions are listed in Supplemental Table S4.

References:

Ahmed I, Islam M, Arshad W, Mannan A, Ahmad W, Mirza B. 2009. High-quality plant DNA extraction for PCR: an easy approach. *J Appl Genet* **50**: 105-107.

Hynes AP, Rousseau GM, Lemay ML, Horvath P, Romero DA, Fremaux C, Moineau S. 2017. An anti-CRISPR from a virulent streptococcal phage inhibits Streptococcus pyogenes Cas9. *Nat Microbiol* **2**: 1374-1380.

Li X. 2011. Infiltration of Nicotiana benthamiana protocol for transient expression via Agrobacterium. *Bio-protocol*: e95-e95.

Schmittgen TD, Livak KJ. 2008. Analyzing real-time PCR data by the comparative C-T method. *Nature Protocols* **3**: 1101-1108.

Song GX, Zhang F, Zhang XW, Gao X, Zhu XX, Fan DD, Tian Y. 2019. AcrIIA5 Inhibits a Broad Range of Cas9 Orthologs by Preventing DNA Target Cleavage. *Cell Rep* **29**: 2579-2589.

Xie M, Muchero W, Bryan AC, Yee K, Guo HB, Zhang J, Tschaplinski TJ, Singan VR, Lindquist E, Payyavula RS et al. 2018. A 5-Enolpyruvylshikimate 3-Phosphate Synthase Functions as a Transcriptional Repressor in Populus. *Plant Cell* **30**: 1645-1660.

Yuan G, Martin S, Hassan MM, Tuskan GA, Yang X. 2022a. PARA: A New Platform for the Rapid Assembly of gRNA Arrays for Multiplexed CRISPR Technologies. *Cells* **11**: 2467.

Yuan GL, Hassan MM, Yao T, Lu HW, Vergara MM, Labbe JL, Muchero W, Pan CT, Chen JG, Tuskan GA et al. 2021a. Plant-Based Biosensors for Detecting CRISPR-Mediated Genome Engineering. *Acs Synth Biol* **10**: 3600-3603.

Yuan GL, Lu HW, De KT, Hassan MM, Liu Y, Li Y, Muchero W, Abraham PE, Tuskan GA, Yang XH. 2022b. An Intein-Mediated Split-nCas9 System for Base Editing in Plants. *Acs Synth Biol* **11**: 2513-2517.

Yuan GL, Lu HW, Tang D, Hassan MM, Li Y, Chen JG, Tuskan GA, Yang XH. 2021b. Expanding the application of a UV-visible reporter for transient gene expression and stable transformation in plants. *Hortic Res-England* **8**: 234.
